# The Impact of Hypoxia in Early Pregnancy on Placental Cells

**DOI:** 10.3390/ijms22189675

**Published:** 2021-09-07

**Authors:** Hui Zhao, Ronald J. Wong, David K. Stevenson

**Affiliations:** Department of Pediatrics, Division of Neonatal and Developmental Medicine, Stanford University School of Medicine, Stanford, CA 94305, USA; huizhao2@stanford.edu (H.Z.); rjwong@stanford.edu (R.J.W.)

**Keywords:** angiogenesis, decidual macrophages, dysregulation, hofbauer cells, heme oxygenase-1, implantation, natural killer cells, placenta, preeclampsia, preterm birth

## Abstract

Oxygen levels in the placental microenvironment throughout gestation are not constant, with severe hypoxic conditions present during the first trimester. This hypoxic phase overlaps with the most critical stages of placental development, i.e., blastocyst implantation, cytotrophoblast invasion, and spiral artery remodeling initiation. Dysregulation of any of these steps in early gestation can result in pregnancy loss and/or adverse pregnancy outcomes. Hypoxia has been shown to regulate not only the self-renewal, proliferation, and differentiation of trophoblast stem cells and progenitor cells, but also the recruitment, phenotype, and function of maternal immune cells. In this review, we will summarize how oxygen levels in early placental development determine the survival, fate, and function of several important cell types, e.g., trophoblast stem cells, extravillous trophoblasts, syncytiotrophoblasts, uterine natural killer cells, Hofbauer cells, and decidual macrophages. We will also discuss the cellular mechanisms used to cope with low oxygen tensions, such as the induction of hypoxia-inducible factor (HIF) or mammalian target of rapamycin (mTOR) signals, regulation of the metabolic pathway, and adaptation to autophagy. Understanding the beneficial roles of hypoxia in early placental development will provide insights into the root cause(s) of some pregnancy disorders, such as spontaneous abortion, preeclampsia, and intrauterine growth restriction.

## 1. Introduction

The placenta is a transient organ established during pregnancy to ensure proper fetal development by providing gas and nutrient exchange between the mother and fetus. Starting from the implantation of the blastocyst, cells originating from the fetus must survive under extremely harsh conditions in the maternal uterus, such as severe hypoxia, lack of vascularization, and a potential attack from the maternal immune system. Therefore, it is required for these cells to aggressively expand to invade uterine tissue and remodel the maternal spiral arteries to develop a vascular network and establish the maternal-fetal interface. Simultaneously, some maternal cells, especially immune cells, are recruited into uterine tissue to regulate vessel remodeling and promote angiogenesis. Thus, the entire process of placental development is a complex, highly regulated process, in which every type of cell needs to dynamically respond to the microenvironment and fulfill their final destiny and function.

Hypoxia has been shown to be associated with several pregnancy disorders. However, the harmful effects of hypoxia mostly occur during mid- to late gestation. In fact, in the early stages of a healthy pregnancy, oxygen (O_2_) availability in the uterus is normally scarce due to low blood circulation. This hypoxic microenvironment is essential for the trophoblast stem cells, or progenitor cells, to maintain homeostasis, prevent DNA damage, and differentiate selectively. Low O_2_ levels are also found in other stem cell niches, such as hemopoietic stem cells (HSCs) and bone marrow (BM), where PO_2_ levels are 9.9–32 mm Hg or 1.3–4.3%, respectively [1], and embryonic stem cells (ESCs) in the blastocyst and embryo compartments. In clinical in vitro fertilization (IVF) protocols, 5% O_2_ is used to culture embryos before implantation. However, recent studies suggest that an optimal O_2_ environment for blastocyst development after day three may require an ultra-low O_2_ level of 2%, which is present in the uterine cavity during peri-implantation [2,3].

Hypoxia also plays a key role in regulating immunity and inflammation. Low tissue O_2_ levels can affect the energy status of immune effector cells and hence suppress immune responses. Hypoxic conditions have also been observed in several physiological as well as pathological immunological niches, such as the BM, lymphoid tissues, placenta, and intestinal mucosa, and tumors and chronically inflamed, infected, or ischemic tissues, respectively [4]. In addition, hypoxia can regulate innate and adaptive immunity by modulating the proliferation, development, and effector function of immune cells, largely via transcriptional changes driven by hypoxia-inducible factor (HIF).

Most studies have been based on human tissue or rodent models. Both human and rodent placentas are hemochorial, characterized by extensive remodeling of the maternal vasculature and direct bathing of the fetal chorion in the maternal blood. In early pregnancy, both species share a similarity for receptivity to blastocyst implantation, endometrial decidualization, uterine artery remodeling, and maternal immune cell composition at the maternal-fetal interface. Even though invasion of trophoblasts into the uterine arteries is much shallower than in the human, rodent models are still used extensively to study the early events of placental development.

## 2. Cells in Early Placental Development

**Figure 1:**
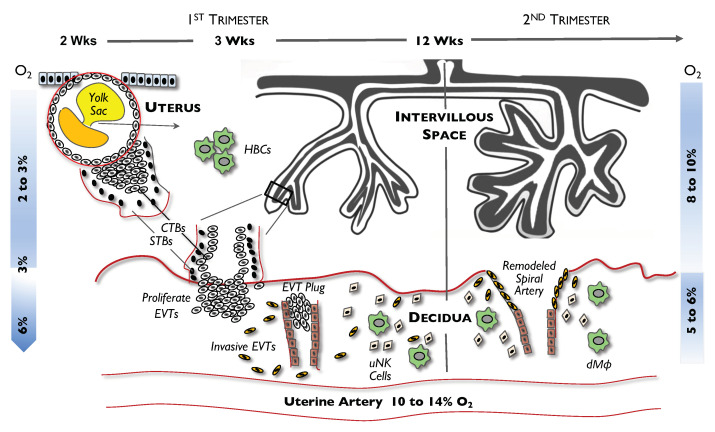
Schematic illustrating placental development—from blastocyst implantation to spiral artery remodeling and villous formation. Different events, such as proliferation of cytotrophoblast (CTBs), differentiation of extravillous trophoblasts (EVTs), formation of the EVT plug in the spiral artery, formation of Hofbauer cells (HBCs), and remodeling of the spiral arteries are shown. The dramatic changes of O_2_ levels in the villous region during the 1st trimester (2–3%) and 2nd trimester (8–10%) compared with the relatively constant O_2_ levels in decidua (5–6%) are highlighted. uNK, uterine natural killer; dMϕ, decidua macrophages; O_2_, oxygen. Reprinted with permission from Ref. [5]. Copyright 2008 Chang C-W, et al.

In pregnancy, fertilization occurs in the fallopian tube within 1–2 days of ovulation followed by the development of the blastocyst in the uterine cavity through day 6 (Figure 1). The blastocyst is formed by the simultaneous development of two distinct layers: (1) the outer trophectoderm and (2) the inner mass endoderm, the latter being the original source of embryonic cells that ultimately develop into a fetus. The trophectoderm is a layer of polarized epithelium cells, or cytotrophoblasts (CTBs), which are normally considered to be trophoblast stem cells. CTBs can proliferate, differentiate, and actively participate in a complex dialogue with maternal cells needed for implantation, anchoring, and invasion, and to ultimately intertwine with uterine tissue to give rise to the placenta.

When the blastocyst adheres to the uterine wall at day 7, CTBs transmigrate across the uterine epithelium and undergo rapid proliferation to expand and establish a CTB column. Cells at the proximal end of a CTB column form a cytotrophoblast shell, from which cells can further differentiate into extravillous trophoblasts (EVTs) at the anchoring villi. Others migrate into the inner third of the myometrium by gaining an invasive ability to establish the placental bed or decidua. EVTs also migrate along the lumen of the spiral arterioles and replace the smooth muscle and the elastic lamina of the vessel wall—an event called “spiral artery remodeling”. CTBs can also fuse and form multinucleated syncytiotrophoblasts (STBs) in the floating chorionic villi, which later enlarge and evolve into the superficial capillary plexus within the endometrium and finally establish the intervillous space or labyrinth.

Like trophoblasts, maternal leukocytes—primarily uterine natural killer (uNK) cells and decidual macrophages (dMϕs)—can also infiltrate into the myometrium. These cells have immunosuppressive and pro-angiogenic properties. In addition, both in vivo and in vitro studies have shown that in the first trimester, uNK cells and dMϕs infiltrate the walls of spiral arteries and mediate the disruption of vascular smooth muscle cells, leading to dilation. These events occur before invasion and relining of the vessels by EVTs. uNK cells can also regulate the timing of EVT migration and the depth of EVT invasion, thereby playing a critical role in spiral artery remodeling.

Different from dMϕs, which are recruited from the maternal circulation, there is another homogeneous population of macrophages of fetal origin, which are called Hofbauer cells (HBCs). These cells are typically found in the endothelium and in proximity to trophoblasts, through which they regulate placental vascular development via paracrine signaling or cell-to-cell crosstalk [6,7]. HBCs secrete a range of pro-angiogenic factors that have a role in vascularization and the remodeling of blood vessels. They are also able to respond to toll-like receptor (TLR) stimulation and are phagocytotic, suggesting that HBCs can have a defensive microbicidal capacity to prevent microbes from entering the placenta.

## 3. Hypoxic Conditions in Early Placental Development

Early gestation is a very critical period for placental development in both humans and rodents. Although many events occur during that period, a notable phenomenon is the exposure of the blastocyst to severe hypoxia (as low as 2–3% or a PO_2_ of 15–20 mm Hg) in the uterus at day 6 post-conception; a similar O_2_ tension is found within the non-pregnant mouse uterus [8,9]. Moreover, after the trophoblast expands and a cluster of EVTs starts to migrate and invade the wall of the uterine spiral arteries, the vessels become plugged, thereby preventing maternal blood from flowing into the intervillous space. As such, a severe hypoxic microenvironment is maintained and can persist for up to 10 weeks of pregnancy [10]. At the end of the first trimester, the plug dissolves and the maternal arterial circulation can now fully enter the intervillous space, subsequently raising and returning the local uterine O_2_ level to its “normal” physiologic level of 8–10% (PO_2_ of 60–76 mm Hg). This process of hypoxic-ischemic/re-oxygenation is normal, physiologic, and essential for normal fetal and placental development [11,12,13].

As described above, hypoxic conditions may not always be harmful to cells, and sometimes are beneficial and protective. In fact, tissue hypoxia is not necessary to induce cellular hypoxia (defined as when the O_2_ supply is not sufficient to meet metabolic demands). Data from mouse studies suggest that O_2_ consumption by embryonic cells in early pregnancy is low, being referred to as “quiet metabolism”, and limits the production of potentially harmful reactive oxygen species (ROS) as well as protects the embryo from free radical-mediated teratogenesis [14]. In addition, the rise in O_2_ levels within the placenta and the embryonic compartments at the end of the first trimester notably overlaps with the conclusion of organogenesis. At this stage of fetal development, the risk of teratogenesis decreases, as differentiation of the major organs is complete.

Normal partial pressures (mm Hg) of arterial O_2_ (PaO_2_) levels of individuals living at various altitudes are as follows: at sea level, PaO_2_ = 75–100; at 4000 m, PaO_2_ = ~50; and at very high mountain altitudes, PaO_2_ = 19–30. Even though low birthweight infants, smaller placental size, and higher rates of preeclampsia have been found in mothers living at high altitudes, there is no direct evidence that their fetuses are more hypoxic than those in mothers living at sea level [15]. This may be due to a number of functional and structural adaptations of the placentas from high-altitude pregnancies. Their placental villi undergo compensatory morphological changes that include increased branching of the capillaries with decreased diffusion distances and changes in villous capillary diameters [16,17,18,19]. This is in agreement with the notion that a common reaction to an exposure to chronic hypoxia is neovascularization, with a significant increase in the number, rather than size, of tissue capillaries. However, minimal modifications have been found in first trimester placentas from mothers living at high altitudes.

## 4. General Cellular Response Mechanisms to Hypoxia

### 4.1. Hypoxia-Inducible Factor (HIF)

When cells are exposed to hypoxia, a primary cellular response is the induction of HIF. HIF is a global transcriptional regulator which controls the expression of more than 1000 proteins by binding hypoxic response elements (HRES) in the gene regulatory region. It also mediates mitochondrial function by regulating the metabolic switch from the process of oxidative phosphorylation to glycolysis via signaling through extracellular adenosine receptors [20] (see below). Thus, HIF can affect many cellular processes during hypoxia, such as angiogenesis, cell migration/invasion, cellular metabolism, and immune cell function.

HIF is a heterodimer protein comprised of two subunits: an alpha subunit with two isoforms, HIF-1α and HIF-2α, and a beta subunit [HIF-β or aryl hydrocarbon receptor nuclear translocator (ARNT)]. While the HIF-β subunit is constitutively expressed and its protein is insensitive to O_2_, the expression of HIF-α is inducible and its protein can be degraded rapidly by proteasomes under normoxic conditions. HIF-1α protein is stabilized under hypoxia via reduced oxygen-dependent prolyl hydroxylation and decreased binding to the von Hippel–Lindau protein (VHL), a master regulator of HIF [21]. HIF-α can subsequently translocate into the nucleus, which leads to an increased expression of pro-survival genes. HIF-α protein has a short turnover time (half-life of only 5 min under ambient air) and can quickly respond to local O_2_ fluctuations.

HIF-1α is highly expressed in both human and mouse placentas [22]. It has been noted that HIF-1α protein levels are tightly regulated by cellular O_2_ concentrations [23], but not well correlated with HIF-1α mRNA levels due to post-translational regulation. In the human placenta, the production of HIF-1α protein is gestational age-dependent, with the highest expression in early pregnancy (~5 weeks) [24]. As pregnancy advances, HIF-1α protein levels gradually decrease, and by week 12, are almost undetectable [24]. Similar data are also found in mouse studies, where HIF-1α protein levels are high at 7–9 weeks of gestation when the placental O_2_ microenvironment is 2–3% (severe hypoxia) [25] and start to decrease when the O_2_ levels gradually increase to 8–10% at 10–12 weeks of gestation [11,26,27]. Interestingly, the transcriptional activity of human HIF-1α mRNA peaks between 14–18 weeks of gestation when reoxygenation occurs. Similar observations have been reported in mouse placentas [27], with higher HIF-1α protein levels found before E8.5, but transcriptional activity upregulated around E9.5–E10.5.

Several knockout (KO) mouse models with deficiencies in genes in the HIF-1 family have been established. Mice deficient in HIF-1α or ARNT have an embryonic lethality by E9.5 and E10.5, respectively [28,29,30,31,32]. ARNT KO embryos have defects in the formation of blood vessels in the yolk sac, branchial arches, and placenta [30,31]. These mice also have defects in implantation at E5.5–E7.5 or in placental development around mid-gestation (E10.5), suggesting that genes in the HIF family play a critical role in placental and embryonic development.

HIF-2α, officially known as endothelial PAS domain protein (EPAS) 1, is also expressed in the placenta [33]. HIF-2α is highly expressed in the human placenta during the first trimester in a normal pregnancy. The loss of the HIF-2α gene as shown in mouse KO models leads to embryonic lethality, which is predominantly due to poor development of the embryonic vasculature and placenta. However, an abnormally increased number of HIF-2α-positive nuclei in STBs have also been reported in pathological placentas *in* the second trimester [34] compared with those from a normal pregnancy. This suggests that an aberrant and persistently high level of HIF-2α in STBs lasting beyond the first trimester may adversely affect pregnancy.

### 4.2. mTORs

Mammalian target of rapamycin (mTOR), a serine/threonine protein kinase, is another sensor that responds to extracellular nutrients and O_2_, and subsequently regulates such processes as intracellular protein homeostasis, mitochondrial function, immune responses, and autophagy, to name a few. mTOR binds with either rapamycin-associated TOR (RAPTOR) protein to form the mTOR complex 1 (mTORC1) or the rapamycin-insensitive companion of mTOR (RICTOR) to form the mTOR complex 2 (mTORC2). Dysregulation of the mTOR pathways has been shown to be associated with several human diseases, e.g., diabetes mellitus, obesity, depression, and some cancers [35,36].

Hypoxia or energy depletion can result in the inhibition of mTOR and suppression of protein translation, a process that is independent of the HIF pathway. Hypoxia inhibits mTOR-mediated protein translation by reducing the phosphorylation of mTOR, resulting in a decrease in ATP consumption to conserve cellular energy [37]. In addition, the mTOR1 signaling pathway affects the transcription of genes involved in amino acid transport, lipid metabolism, and immunomodulation [38]. In mice lacking in either mTOR or RAPTOR, fetuses abort around the peri-implantation period, while those with a RICTOR mutation perish later, at ~E10.5, suggesting the important but unique roles of mTORC1 and mTORC2 in early placental and embryonic development [39,40,41]. In addition, inhibition of mTOR activity in human term STBs, treated by either rapamycin or hypoxia, has been found to induce autophagy, a survival process for cells [42].

### 4.3. Metabolic Changes

Low O_2_ levels can prompt cells to switch their metabolic status via HIF-1α from oxidative phosphorylation to anaerobic glycolysis, particularly when cells are exposed to prolonged hypoxia. During oxidative phosphorylation, ATP is generated using O_2_ by the electron transport chain in mitochondria, while during anaerobic glycolysis, ATP, pyruvate, and NADH is produced in the cytosol [26,43,44]. Hypoxia also reduces the use of ATP by downregulating protein translation and the activity of Na^+^/K^+^-ATPase and, in turn, partially reduces ATP production by decreasing the activity of the electron transport chain in mitochondria. This reduced homeostasis is normally referred to as “quiet metabolism”, compared with “noisy metabolism”, where more O_2_ is consumed [45].

A benefit of “quiet metabolism” is the prevention of the overproduction of ROS through the reduction of the respiratory rate in mitochondria. ROS can damage various biomolecules, resulting in lipid peroxidation, protein carbonylation, and DNA strand breakage. Studies on embryogenesis and tissue engineering have shown that “noisy metabolism” is more likely to induce DNA damage [46].

Adverse effects of ROS on stem or progenitor cells have been well-documented [47,48]. Overproduction of ROS can abrogate certain HSC functions, such as cell cycle quiescence, self-renewal, survival, and multi-lineage differentiation capacity. If the levels of antioxidant proteins, superoxide dismutase (SOD), glutathione peroxidase (GPx), catalase (CAT), and heme oxygenase-1 (HO-1) fail to counteract ROS-induced cellular damage, the HSC pool will be reduced via apoptosis and cell cycle activation. Transplanted HSCs in circulation have been shown to have elevated levels of ROS before homing to BM [49]. Fortunately, the “normal” hypoxic environment within the BM limits ROS production such that transplanted HSCs are able to survive.

Reduced ATP production under physiological hypoxic conditions can also suppress inflammatory responses in the placenta. Extracellular ATP has been referred as a “danger-associated molecule” that can initiate and prolong immune responses in an infection-free environment. Pregnant rats administered ATP displayed a preeclampsia-like syndrome, such as hypertension, proteinuria, and general inflammation. ATP can be hydrolyzed by enzymes, e.g., CD39, alkaline phosphatase, and CD73, into ADP and AMP, which subsequently are degraded into adenosine. Adenosine can counteract ATP-induced effects, since adenosine suppresses neutrophil and monocyte/macrophage activation and recruitment, thus inducing tolerance of a T-cell response. Elevated adenosine levels have been found in plasma from pregnant women, and adenosine receptors (A_2A_ and A_2B_) as well as enzymes that degrade ATP to adenosine have been found to be expressed in trophoblasts. Hypoxia can also increase the expression of A_2A_ receptors in placental explants from women who delivered a normal pregnancy, but not in those women with preeclampsia. All these findings suggest that ATP, adenosine, and their receptors play an important role in maintaining a healthy pregnancy [50] and may mediate immune tolerance through T cells [51,52]. 

On the other hand, extensive proliferation and sustenance of trophoblast cells in early pregnancy requires an abundant energy source. Even though anaerobic glycolysis provides an effective means to generate carbon skeletons for the biogenesis of amino acids and lipids needed for rapid cell proliferation, less ATP is produced than in oxidative phosphorylation. Hence, cells may need to accumulate more glucose to compensate, as seen in expanding cancer cells in a hypoxic niche [53]. When tissue glucose is abundantly available, ATP production is substantially faster using glycolysis than oxidative phosphorylation [54,55]. Considering that glucose transporters (GLUT family of transporters) can be upregulated by HIF-1 and that hypoxic cancer cells have increased GLUT expression and a 10- to 100-fold increase in glucose levels, we speculate that GLUT expression and glucose abundance may also be upregulated in highly proliferated trophoblast cells in early pregnancy [43,53,56].

### 4.4. Autophagy

Autophagy is a survival strategy that allows cells to overcome various stressors, e.g., nutrient starvation, hypoxia, growth factor withdrawal, and chemotherapeutic stress. It is a key catabolic process involved in the elimination and recycling of bulk cytoplasmic constituents, damaged proteins, and intracellular organelles through lysosomal proteolysis. At initiation, an isolation membrane (phagophore) inside the cytoplasm is formed, then stretches into an autophagosome. The mature autophagosome then surrounds intracellular cargo (i.e., mitochondria, protein aggregates, and lipid droplets) and also microorganisms that have attacked the host cell. The resulting autophagosome fuses with lysosomes to form autophagolysosomes, where the autophagosomal cargos are degraded by lysosomal proteases. The benefits for cells undergoing autophagy include limited nutrient usage, minimized ROS buildup, and clearance of misfolded proteins.

Autophagy can be activated via different signaling pathways (such as HIF and mTOR) depending on the degree of cellular exposure to hypoxia or anoxia. In brief, HIF-dependent signaling after exposure to 1–3% O_2_ abates beclin 1 (Bcl-1)/Bcl-2 interaction, resulting in autophagy; whereas under anoxia (<0.1% O_2_), HIF-independent signaling induces autophagy via adenosine 5′ monophosphate-activated protein kinase (AMPK) or the unfolded protein response pathway. Because O_2_ levels in the first trimester uterus range from 1–3%, HIF-dependent pathways are speculated to contribute to autophagy in placental cells in early gestation.

Dysregulation of autophagic activity has been associated with human diseases, such as neurodegenerative diseases, infection/inflammation, and cancers. One notable example is Alzheimer’s disease, in which brain cells have an increased accumulation of autophagosomes due to impaired autophagic activity [57]. Upregulated autophagy has also been observed in cancer cells after exposure to local stressors, e.g., severe hypoxia and limited blood supply. Those conditions are very similar to the microenvironment found in early placental development. In fact, increased autophagy, assessed by the expression of LC3-II and p62, has been found in primary cell cultures of human trophoblasts under hypoxic conditions. Placentas from women with preeclampsia or fetal growth restriction (FGR) have increased autophagic activity in villous trophoblasts compared with placentas from normal pregnancies [58,59].

### 4.5. Epigenetic Alterations and miRNA Function

Hypoxia may regulate epigenetic changes and microRNA (miRNA) expression in placental cells, directly or indirectly, and subsequently alter gene expression. DNA methylation, the most extensively studied epigenetic change, is characterized by methylation of cytosine residues next to 5’ guanine (CpG dinucleotides). Since DNA hypomethylation induces gene transcription while DNA hypermethylation leads to transcriptional repression, the global DNA methylation status in cells therefore could be the determining factor for the ultimate destiny of a cell. As early as day 5–6 post-fertilization, the trophectoderm in the blastocyst is hypomethylated while the inner cell mass is hypermethylated. In murine models, hypomethylation of E74-like factor 5 (Elf5) and placenta-expressed transcript 1 (Plet1) promoters in trophoblast cells impacts the establishment of their stemness state and their self-renewal [60]. Reduced methylation levels are found from trophoblastic tissues derived from chorionic villous samples during the first trimester of pregnancy. In addition, DNA hypomethylation also allows CTBs to transition to invasive and migratory EVTs [61,62]. This relative hypomethylation status reflects the intense transcriptional activities required for the highly proliferative and invasive nature found in the early placental development.

Along with trophoblast differentiation, trophoblast stem cells, including CTBs, EVTs, and STBs, have distinct profiles of DNA methylation as well as miRNA expression, especially in genes that are involved in cell cycle regulation and differentiation and the regulation of pluripotency in both rodents and humans [62,63,64]. The most direct evidence of an effect of O_2_ levels on DNA methylation and gene expression from human villous CTBs and STBs were shown in studies performed by Yuen et al. [65]. They exposed primary cell cultures of human CTBs and STBs with <1%, 8%, and 20% O_2_ and found no changes in the average DNA methylation status but observed that a set of loci became hypermethylated only in CTBs, suggesting the effect of hypoxia may be cell type-dependent. Moreover, these changes seem to be associated with the transcriptional factor AP-1, which is triggered by hypoxia and can interact with DNA methyltransferases to target methylation at specific sites in the genome. On the other hand, the DNA methylation state may affect cellular responses to hypoxia. Due to a CpG dinucleotide found in the consensus sequence of HIF-1 binding sites, it is suggested that the expression of some HIF-1-dependent genes is also epigenetically regulated [66]. Actually, CpG dinucleotides are mostly hypomethylated in the HIF binding site of genes for developmental and stress response (hypoxia and inflammation) factors [67]. Pregnant rats treated with methylation inhibitors or DNA methyltransferase KO mice are shown to have placental defects, including smaller size, abnormal structure, and improper ratios of trophoblast populations [68]. 

miRNAs are small non-coding single stranded RNAs that can modify translation by changing mRNA stability. miRNAs can be upregulated under hypoxia and, in turn, are actively involved in cellular responses to hypoxia. Those miRNAs that are induced under direct regulation of hypoxia-induced transcription factors are called ‘hypoxamirs’. For example, miR-210, one of the most extensively studied hypoxamirs, is upregulated directly by HIF-1α and capable of inhibiting migration and invasion in primary CTBs and EVTs. miR-210 also regulates genes related to mitochondrial function and angiogenesis activity [69]. However, several miRNAs, such as miR-155, miR-21-5p, and miR-23-3p, can downregulate VHL tumor suppressors and consequently modify HIF-1α stability. Hypoxia can also influence miRNA biogenetic components, such as Drosha (a class 2 ribonuclease enzyme) and the riboendonuclease Dicer [70]. miRNAs have been found in placental cells, especially in trophoblast cells, and play a pivotal role in regulating placental development and function under hypoxic environments. miRNAs can also promote maternal immunotolerance by regulating human leukocyte antigen-G (HLA-G) expression via miR-148a, miR-152, and miR-365 [69].

## 5. Effect of Hypoxia on Placental Cells

### 5.1. Hypoxia on CTB Expansion

During the period of blastocyst formation and peri-implantation, O_2_ levels in the uterus are extremely low but vary depending on the location. In 1996, Fisher and colleagues [12] found that in the primate uterus, the O_2_ levels in the oviduct and fallopian tubes are ~5% but drop to ~2%. Therefore, it is under this severe physiologic hypoxia that CTBs inside the blastocyst actively proliferate and expand to finally form a CTB shell within the uterus. As mentioned above, due to metabolic changes, hypoxia can promote the characteristic of “stemness” in stem cell populations and maintain pluripotency before differentiation [71].

In general, mice deficient in genes involved in hypoxic signaling (HIF, mTOR) and in endoplasmic reticulum (ER) stress have defects during the peri-implantation period (E5.5–E7.5) when the majority of pregnancy losses occurs, or around mid-gestation (E10.5), suggesting difficulties in CTB pool maintenance or, later, in trophoblast differentiation. However, if CTBs are exposed to a lower O_2_ level than those in the uterus (2–3%), detrimental effects would also be induced. Yang Y et al. [72] cultured mouse CTBs in 0.5% O_2_ for more than 1 day and found decreased proliferation, diminished potency and stemness, and increased apoptosis and differentiation. All effects were irreversible and associated with adverse pregnancy outcomes.

The aggressive proliferation of CTBs in early pregnancy is believed to be crucial and beneficial for both placental and fetal development. The rapid expansion of CTBs provides a reservoir of stem cells that can later differentiate into either EVTs or STBs. It is also interesting to note that although they are derived from the same blastocyst, the mass of placental cells increases much faster than the embryonic cells in the first half of pregnancy. Therefore, the placenta is established earlier to accommodate for a later growing embryo with the necessary gases and nutrients from a mother.

### 5.2. Effects of Hypoxia on Trophoblast Differentiation

Depending on the signal, mononucleated CTBs differentiate into either EVTs or multinucleated STBs. Based on extensive research using different models—rodents (both mouse and rat), human trophoblast cell lines, and primary human villous explants—it is now widely accepted that the O_2_ microenvironment contributes significantly to cell differentiation and cell destiny [5,73,74].

#### 5.2.1. EVT Differentiation

CTBs from the cytotrophoblast shell start to differentiate into invasive EVTs early in the first trimester, when the local O_2_ levels are 2–3%. When transitioning from an epithelial to a mesenchymal phenotype, EVTs lose p63 and epidermal growth factor receptor (EGFR) (cell surface markers on CTBs) and express HLA-G. When culturing first trimester human villous explants onto a 3D extracellular matrix, Jauniaux et al. [75] found that the production of EVTs is triggered only after exposure to 2–3% O_2_, but not to 20% O_2_. These EVTs then adopt a pleiotropic phenotype and mature as they migrate to the decidua, where the O_2_ level is higher. This suggests that the differentiation of EVTs takes place under low O_2_ levels, while its maturation occurs under relatively higher O_2_ conditions. Insufficient EVT differentiation could lead to the formation of a poor EVT plug in the spiral artery, which would lead to an unobstructed entry of the maternal circulation into the intervillous spaces and a premature increase in O_2_ levels, which can then induce oxidative damage to placental cells [44,76].

Several studies have suggested that low O_2_ levels can also promote EVT invasion. For example, when pregnant rats inhale 11% O_2_ post-implantation, EVT invasion was enhanced, resulting in a robust expansion of the mesometrial vessels [77]. In addition, using a pregnant rhesus monkey model, Zhou et al. [78] reduced uteroplacental perfusion by placing a stricture around the abdominal aorta to create an “hypoxic” placenta, and found that interstitial EVT invasion was greatly enhanced compared with controls. Insufficient EVT invasion could result in poor spiral artery remodeling. Since the dilation of arterial vessels is directly related to blood flow velocity, a less dilated vessel will produce a higher flow shear rate of maternal blood and cause structural damage of the intervillous vascular network [79]. Clinically, the retention (or presence) of smooth muscle within the vessel walls is a hallmark of early-onset preeclampsia and FGR [80,81]. On the other hand, an overaggressive EVT invasion into the myometrium is also harmful, being the major cause of placenta accreta.

The presence of the HIF complex is required during EVT differentiation and invasion. HIF-1α is present in CTBs and EVTs in the human placenta during early gestation, and its expression has been found to be increased in CTBs that transition to EVTs in both in vivo and in vitro experiments [82], suggesting that hypoxia is required for EVT differentiation. It has been reported that pregnant HIF-1α KO mice can reproduce normally, with litter sizes similar across genotypes, while the disruption of either the HIF-1α or Arnt subunit in fetal trophoblasts results in chorion/allantois fusion, an abnormal labyrinth layer, and a diminished junction zone (spongiotrophoblast and trophoblast giant cells, the equivalent of EVTs in rodents) [83,84]. Moreover, in knockdown studies of the HIF complex (Arnt^−/−^), trophoblast stem cells failed to differentiate into EVTs, but only exclusively into labyrinth intravillous trophoblast cells (or STBs) [83,84]. This abnormal switching in the trophoblast lineage is mediated by changes in histone acetylation and has been replicated by treating wild-type (WT) trophoblast stem cells with histone deacetylase inhibitors during differentiation [85]. Moreover, hypoxia also confers placental immune “privilege”, where maternal immune cells, such as uNKs and dΜϕs, are prevented from attacking fetal-derived semi-allogeneic EVTs in the decidua [86,87]. Via the HIF-1α pathway, hypoxia induces the expression of HLA-G, a non-classical major histocompatibility complex (MHC) class I antigen on the surfaces of EVTs, to create “immune invisibility” and induce immunotolerance.

#### 5.2.2. STB Differentiation

STBs are one of the main cells that constitute the placental intervillous capillary structure, which forms the fetoplacental barrier; therefore, these cells play a critical role in maternal-fetal nutrient and gas exchange. STBs can also secrete several hormones for pregnancy maintenance, such as human chorionic gonadotropin (HCG) and human placental lactogen (HPL).

Differentiation of mononuclear CTBs into multinucleated STBs takes place in a relatively high O_2_ microenvironment. In studies where human trophoblasts were cultured in vitro under different O_2_ levels, they found that a level of 21% promotes spontaneous CTB cell fusion into STBs, while a lower O_2_ level (<11%) significantly reduced cell fusion and downregulated hormone levels secreted by STBs [82,88,89]. The inhibition of STB differentiation under the hypoxic conditions present in the first trimester partially relies on an intact HIF complex. In the HIF-mutant mouse, CTBs differentiate exclusively into STBs [85], suggesting that without a hypoxic environment or stimulus, CTBs become STBs by default. A defect in HIF-1β (ARNT) in primary CTBs can restore STB-produced HCG secretion [82]. A recent report by Albers et al. [90] showed that a prolonged expression of trophoblast-specific HIF-1α, which mimics an extended hypoxic condition, beyond the first trimester can lead to significant detrimental effects on vascular development, such as a reduction in branching morphogenesis, alterations in the intervillous spaces, and a failure to remodel spiral arteries, and can also result in preeclampsia-like symptoms and FGR.

### 5.3. Hypoxia and uNK Cells

uNK cells are the most abundant lymphocyte population present at the maternal-fetal interface in both human and murine pregnancies [91,92]. They are a heterogeneous population and peak in frequency during the first trimester, constituting 50–90% of total leukocytes. They then progressively decrease in numbers and granularity from mid-gestation to term. The origin of these specialized NK cells has always been intriguing. In both human and murine pregnancies, uNK cells have been found in the endometrium pre-pregnancy, and then markedly expand, becoming gradually more granulated during the progesterone-dominated secretory phase post-ovulation and throughout the first trimester. These uNK cells can be replenished during each menstrual cycle, and therefore are suspected to be tissue resident NK (trNK) progenitor cells. It was also proposed that the initial proliferation of local trNKs in the first trimester is followed by the recruitment of circulating NK (cNK) cells in mid-gestation [91]. However, Chakraborty et al. [93] administered anti-asialo GM1, a NK cell-depleting antibody, to pregnant rats at E4.5 and E9.5 and found complete depletion of uNK cells at E9.5 and E13.5. Moreover, by analyzing donor- and recipient-specific HLA molecules expressed on uNK cells from women who had undergone uterine transplantations and later achieved successful pregnancies, Strunz et al. [94] reported that all uNK cells in transplanted uteri have only recipient markers, indicating they were cNK cells from the circulation. The proliferation of these cNK cells coupled to differentiation into uNK cells is shown to be mediated through IL-15 signaling [94]. These studies suggest that uNK cells are fully dependent on the recruitment of cNK cells from the maternal circulation, but these recruited cNK cells might be NK progenitor cells [93,94].

Generally, NK cells are preferentially found in the most hypoxic areas compared with other immune cells, such as T and B cells [95]. Even though the O_2_ levels in the endometrium and myometrium are not as low as those in the implantation sites during early pregnancy, migrating cNK progenitor cells to uterine tissue (where PO_2_ is 15–25 mm Hg) are still exposed to hypoxic conditions compared with those present in the arterial circulation (where PO_2_ is 75–100 mm Hg). It has been reported that a deficiency of HIF in maternal sites can induce less recruitment of uNK cells into the maternal decidua. Once migrating to the uterus, they become highly proliferative, as shown by increased BrdU incorporation and high Ki67 expression. This phenomenon further supports the notion that progenitor cells generally exhibit higher rates of proliferation and can better retain their multipotent state in a hypoxic environment.

The phenotypes and functions of uNK cells acquired in the uterus may relate to its hypoxic microenvironment. NK cells assume a more immunosuppressive (versus a cytotoxic) function under hypoxic conditions [96,97,98,99]. Although NK cells are best known for their cytotoxic properties (killing virus-infected cells and surveilling for early cancer cells), mature uNK cells, which are large with granules encasing perforin and granzymes, are poorly cytotoxic and produce less inflammatory cytokines. Instead, they express angiogenic factors, growth factors, and adhesion/matrix proteins, which in turn regulate angiogenesis, endovascular invasion, and spiral artery remodeling [93].

The functional adaptation of uNK cells to hypoxia is believed to be mediated by HIF. Krzywinska et al. [100] have shown that the deletion of HIF-1α in NK cells increases the bioavailability of vascular endothelial growth factor (VEGF) by decreasing the expression of soluble VEGR receptor-1 (sVEGFR1). When these HIF-1α-null NK cells infiltrate into solid tumors where hypoxia is present, the vascular network of the tumor is adversely affected, which is characterized by a high density of immature vessels, areas of severe hemorrhage and increased hypoxia, and enhanced metastasis due to non-productive angiogenesis [100].

On the other hand, uNK cells can also affect the O_2_ environment in the placental intervillous spaces by regulating EVT invasion and spiral artery development. Using a rat model, Chakraborty et al. [101] found that depleting NK cells at the maternal-fetal interface from E4.5–E8.5 results in delayed spiral artery remodeling and subsequently induces transient hypoxia from E8.5–E9.5. This critical gestational interval also represents a key developmental phase associated with the switching of the differentiation of CTBs from EVTs to STBs, subsequently regulating the development of the decidua and junctional zone versus the labyrinth zone, respectively. Therefore, the absence of uNK cells or a maternal hypoxic environment during E4.5–E8.5 can result in a deeper invasion of EVTs into the spiral arteries and abnormal expansion of the junctional zone relative to the labyrinth zone [101].

### 5.4. Effect of Hypoxia on Macrophages

Placental macrophages are another heterogeneous population found in the placenta throughout gestation. They orchestrate a broad spectrum of biological functions, such as microbicidal activity, angiogenesis, and phagocytosis of apoptotic cells. Although placental macrophages have high plasticity and heterogeneity, two main groups have been identified based on their origin: (1) residential macrophages, which originate from the fetus, and are usually referred to as HBCs (human CD14^+^HLA^−^DR^−^FOLR2^+^, mouse CD11bloF4/80^hi^Cx3CR1^hi^ as described previously and below). They are actually the only fetally-derived immune cell population present within the stroma of a healthy placenta; and (2) macrophages replenished from the maternal circulation by HSC-derived cells (human CD14^+^HLA^−^DR^hi^, mouse CD11b^hi^F4/80^lo^) [102] found in the decidua throughout gestation.

#### 5.4.1. HBCs

HBC progenitors have clonogenic properties, emerge as early as 18 days post-fertilization [103,104], and are present in the chorionic plate of the placenta before the onset of the fetoplacental circulation. Later, mature HBCs migrate to the villi and remain throughout gestation [105]. Their initial appearance in the placenta is thought to be mediated via a process called “primitive hematopoiesis”, which occurs only within the yolk sac in mice but takes place in both the human yolk sac and placenta [106]. In the first trimester, placental HBCs are found to be transcriptionally similar to macrophages in the yolk sac, suggesting that they might have the same origin. Unlike the differentiation of HSCs to macrophages, HBC progenitor cells proliferate in situ and mature directly into HBCs without monocytic intermediates [107,108]. Since primitive embryonic hematopoiesis takes place under severe hypoxic conditions, it is speculated that low O_2_ levels could also be a critical factor for the normal expansion and development of placental HBC progenitors in the first trimester [106,109,110].

Limited access to human placental tissues in early gestation has hindered our understanding of the effects of hypoxia on HBC development. Instead, studies using embryonic cells might provide some crucial insights. Cipolleschi et al. [111] isolated myelo-erythroid colonies, which are erythroid burst-forming units containing the progenitors for yolk sac macrophages, from human cord blood and then cultured these colonies under severe hypoxia (1% O_2_). They found that under this condition the maintenance and cloning efficiencies are enhanced. Adelman et al. [112] used Arnt^−/−^ (null) mutants to demonstrate that embryonic multilineage hematopoietic progenitors are also regulated under hypoxia via a HIF complex. Embryoid bodies with Arnt KO mutants were unable to proliferate, and the numbers of hematopoietic progenitors in the yolk sac were significantly decreased.

HBCs phenotypically and functionally resemble alternatively activated macrophages (M2) and have broad roles in placental morphogenesis and homeostasis. They can produce high levels of VEGF and sprouty proteins to modulate placental villous branching and tissue remodeling [113]. HBCs also migrate to the villi and assist in the terminal maturation of primitive embryonic red blood cells. In addition, they have a microbicidal capacity since they can respond to TLRs [102]. Alterations of HBCs have been associated with several pregnancy disorders [113]. In patients with severe preeclampsia, the numbers of HBCs and their expression of dendritic cell-specific intercellular adhesion molecule-3-grabbing non-integrin (DC-SIGN) and IL-10 are significantly reduced, while in women with HELLP syndrome, the number of HBCs are increased. In addition, HBCs are significantly reduced in chorioamnionitis and may contribute to preterm birth. Furthermore, when HBCs undergo hyperplasia or proliferation in chronic villitis caused by infection with cytomegalovirus, Zika, herpes simplex viruses, and others, placental damage may occur [102,114].

#### 5.4.2. dMϕs

Unlike HBCs, macrophage populations found in the placental bed (decidua) primarily arise from the maternal peripheral circulation—from monocytes or myeloid progenitor cells. After migrating to the uterus, monocytes respond to cues in the microenvironment and transform into specific phenotypes. There are conflicting reports about phenotypes of dMϕs, which is possibly due to the differences in isolation techniques used in a particular study or in collection times of the decidua. dΜϕs are found near the spiral arteries and can secrete cytokines, growth factors, angiogenic factors, and hormones, suggesting their role in spiral artery remodeling, apoptotic cell phagocytosis, recruitment and crosstalk of other decidua cells, and coordination of the immune response [115].

The data on the effects of the O_2_ environment on dMϕ function are limited, such that the mechanism remains elusive. dMϕs display some similarity with tumor-associated macrophages (TAMs) [116], which are also recruited from the circulation and located in hypoxic niches (0.1–5% O_2_) within solid tumors [117,118]. Hypoxia is now recognized as a frequent and important feature of rapidly growing and aggressive solid tumors. The mechanism of how TAMs respond to hypoxia may provide clues to what is happening to dMϕs. Studies on TAMs have shown that low O_2_ levels in tissues significantly promote recruitment of monocytes and myeloid-derived suppressor cells (MDSCs) into local sites and subsequently induce pronounced T-cell inhibitory effects [119]. Hypoxia also shapes and induces specific macrophage phenotypes that promote tumor malignancy, as hypoxia fosters immune evasion, angiogenesis, tumor survival, and metastatic dissemination. It is also involved in the regulation of MHC expression in tumor cells to induce “immune invisibility”, which is very similar to the expression of HLA-G on EVTs, as mentioned above.

## 6. A Mouse Model Representing a Failure to Response to Hypoxia

Animals with mutations in the genes or pathways that directly respond to hypoxia (“first responders”) have produced valuable insights and contributed tremendously to our understanding of the impact of hypoxia on placental development. Among them are gene KO rodent models in the HIF family/pathway and mTOR family/pathway, as we have described above. Moreover, HIF and mTOR families can further regulate the expression of over a thousand genes that mediate many downstream cellular responses, such as angiogenesis, migration/invasion, erythropoiesis, and cell metabolism. Therefore, we hypothesized that improper expression of those downstream genes would adversely affect placental development. One example is genes in the VEGF-VEGFR family, whose expression is under control of HIF-1 and whose function is related to placental angiogenesis. Mutations in VEGF-A, PIGF, VEGFR1, and VEGFR2 genes, some affecting even only a single alle, can cause severe vascular defects in the embryo and placenta and lead to fetal death in the early stages of pregnancy [120,121].

A pregnant HO-1-deficient mouse may represent another model to study placental defects due to the improper response of cells to hypoxia. We have shown that placentas from pregnant HO-1^+/−^ (HO-1 Het) mice have vascular defects in early gestation. HO-1 is the inducible HO isozyme that degrades heme to produce carbon monoxide (CO), ferrous iron (Fe^2+^), and biliverdin/bilirubin [122,123]. Even though its expression is not directly induced by O_2_ levels, HO-1 can be transcriptionally upregulated by HIF-1α. The role of HO-1 in maintaining cellular homeostasis is reported to be mediated via mTOR signaling. The HO-1/CO pathway can also affect mitochondrial biogenesis and energy transfer, which in turn controls ROS production, cellular metabolism, and autophagy. The anti-inflammatory property of HO-1 has a reciprocal relationship with the ATP/adenosine/receptor signaling pathway [124,125], and it may be an important therapeutic target in inflammatory diseases. In addition, Cao et al. [126] demonstrated that in HO-1 deficiency, the balance between HSC self-renewal and differentiation is perturbed in the hypoxic BM. These data suggest that HO-1 is actively involved in the regulation of major cellular mechanisms in response to hypoxia.

**Figure 2:**
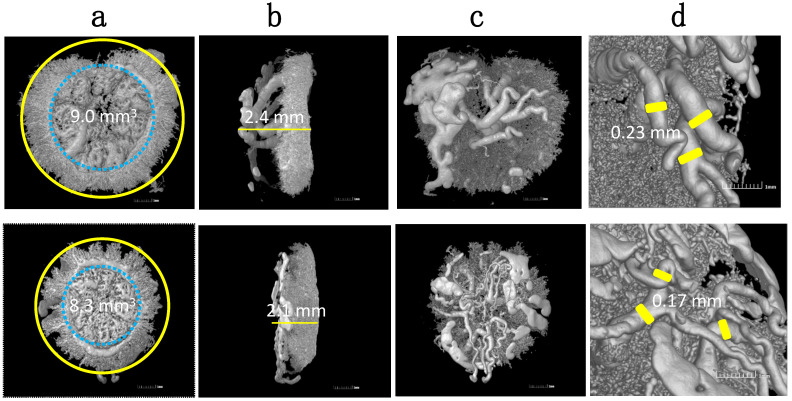
Representative 3D vascular castings of HO-1 Het (top panel) and WT (bottom panel) placentas harvested at E16.5 using MicroCT. Images show differences in: (**a**) circumferences; (**b**) thicknesses; (**c**) maternal vasculatures; and (**d**) spiral artery diameters. Reprinted with permission from Ref. [129]. Copyright 2011 Zhao H, et al.

HO-1 is found to be expressed in the ectoplacental cone (as the expanded CTB column in humans) as early as E6.5 and then in the junction zone, which includes spongiotrophoblasts (human EVT region) when trophoblasts invade into the uterus. HO-1 KO (HO-1^−/−^) embryos do not survive through early pregnancy–normally aborting before E8.5. In HO-1 Het pregnancies, viable offspring are delivered, but are growth restricted, with the mother displaying preeclampsia-like features, such as hypertension and high soluble fms-like tyrosine kinase-1 (sFLt-1) levels [127]. Our studies using histochemistry further confirmed that HO-1 Het placentas have a markedly thinner junction zone and malformed vascular structures in the labyrinth compared with WT placentas [127]. By using a vascular corrosion casting technique and 3D reconstruction of MicroCT images, we were able to visualize the vasculature of a HO-1 Het placenta and demonstrated insufficient spiral artery remodeling (Figure 2) [128,129]. In addition, fewer numbers of uNK cells, altered uNK differentiation and maturation, and lower expression of uNK-related angiogenesis factors were also observed in HO-1 Het deciduas [128,129,130]. Recently, we reported that HO-1 was highly expressed in myeloid cells, and the recruitment of dMϕs was significantly reduced in HO-1 Het placentas [131]. All these changes demonstrate that HO-1 plays a pivotal role in regulating the phenotype and function of various placental cells, including trophoblasts, uNK cells, and dΜϕs.

**Figure 3:**
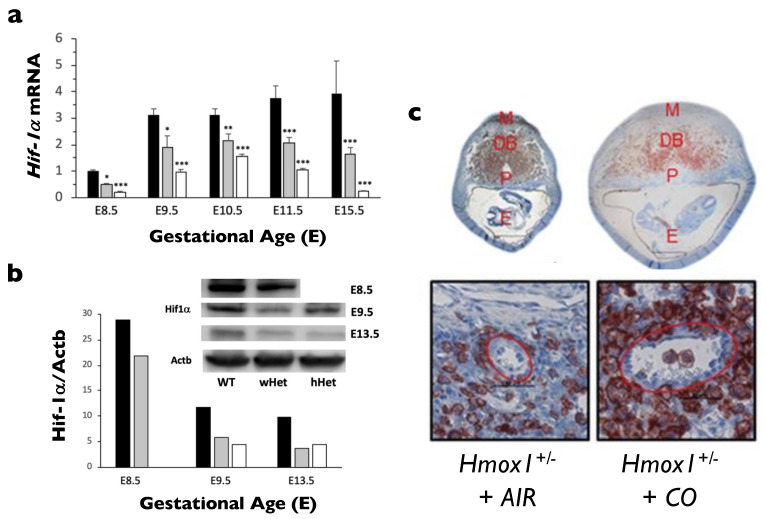
Relationship between HO-1 and HIF-1α. (**a**) *HIF-1α* mRNA expression was measured in placentas at various gestational ages harvested from WT pregnancies (WT, black bars, *n* = 3 at each age), HO-1 Het pregnancies with WT fetuses (wHet, gray bars, *n* = 3 at each age) or HO-1 Het fetuses (hHet, white bars, *n* = 3 at each age). *HIF-1α* expression was normalized to *Gapdh* and *Actb* levels. (**b**) HIF-1α protein levels in placentas at various gestational ages harvested from HO-1 Het pregnancies with WT fetuses (wHet, gray bars) or HO-1 Het fetuses (hHet, white bars) compared with placentas from WT pregnancies (black bars) are shown by Western blots. HIF-1α expression was normalized to Actb levels. Due to the extremely small size of fetuses at E8.5, standard genotyping could not be performed. Therefore, *Hmox1* mRNA levels were used to differentiate wHet and hHet placentas for mRNA studies, but this was not feasible for Western blots. * *p* < 0.05; ** *p* < 0.01; *** *p* < 0.005. Reprinted from Zhao H, et al., *Placenta* **2020**, *99*, 108–116, no permission required. (**c**) Administration of 50 ppm CO to pregnant HO-1 Het mice (*Hmox1*^+/-^ + CO) at E3 to E8 increased the abundance of uNK cells (samples collected at E10) and improved spiral artery remodeling compared with placentas from pregnant HO-1 Het mice exposed to ambient air (*Hmox1*^+/-^ + AIR). DBA lectin staining of mid-sagitally cut whole implantation sites (top panel, DB, decidua basalis; E, embryo cavity; M, MLAP; P, placenta) and representative images of spiral arteries at the fetomaternal interface (400×; scale bar: 50 µm). Reprinted with permission from Ref. [130]. Copyright 2014 Linzke N, et al.

Interestingly, some abnormal features of the HO-1 Het placenta closely resemble those of the HIF-1α-deficient placenta, leading us to speculate that HO-1 deficiency may contribute to the improper response of CTBs to hypoxia and subsequently alter EVT differentiation and invasion. Indeed, we found that both HIF-1α mRNA and protein levels were significantly lower in HO-1 Het placentas than those in WT placentas throughout gestation (Figure 3a,b). Moreover, HIF-1α transcription was lower in HO-1 Het and KO macrophages compared with those of the WT, clearly indicating a reciprocal relationship between HIF-1α and HO-1 [27]. More interestingly, only WT placental lysates harvested from first trimester pregnancies, but not those from an HO-1 Het or the 2nd-trimester pregnancies, can restore HIF-1α expression in HO-1 Het placental cells, implying that some factors in the first trimester WT placental lysates might be able to overcome HIF-1α dysfunction observed in HO-1 deficiency [27]. In support of our finding, Linzke et al. [130] exposed low doses of CO (50 ppm) to pregnant HO-1-deficient dams from days 3–8 of gestation and successfully reversed some placental defects (Figure 3c). CO exposure not only improved abnormal spiral artery remodeling and high blood pressures, but also enhanced the in situ proliferation of uNK cells and normalized the angiogenic parameters, subsequently restoring fetal growth. As CO inhalation reduced tissue O_2_ levels and thus promoted more CTB to EVT differentiation, the finding may explain why CO generated from tobacco smoking in pregnant women can reduce the onset of preeclampsia and gestational hypertension [132,133].

Gene KO animal models have provided good examples of how the “first responder” genes to hypoxia, such as HIF and mTOR, and those downstream genes, such as VEGF and HO-1, can work in unison in the cellular adaptation to hypoxia, such that a dysregulation in any of these pathways could lead to pregnancy disorders. Further research is needed to identify more downstream genes that can affect placental cells to respond to a hypoxic microenvironment. These studies will improve our understanding of the effects of hypoxia on the early stages of placental development.

## 7. Conclusions

Although it is commonly believed that a hypoxic microenvironment causes adverse effects on pregnancy and embryonic development, it is actually present in a healthy uterus, placenta, and embryo in early gestation. Hypoxia can reduce ROS production and limit DNA damage and mutations in stem or progenitor cells, can control the fate of differentiated trophoblast cells, and can induce immunosuppression and tolerance of maternal immune cells to semi-allogeneic fetal-derived cells. Hypoxia is therefore very critical and essential for the establishment of a healthy pregnancy, regulating many hallmark events, such as blastocyst implantation, trophoblast cell anchoring, decidual development, spiral artery remodeling, immune tolerance, and angiogenesis/vasculogenesis. Therefore, hypoxia-sensing molecules or pathways (HIF and mTOR), as well as the cellular adaptive mechanisms to hypoxia (metabolic switch and autophagy), may play pivotal roles in early placentogenesis. Any genetic mutations that directly or indirectly cause an inadequate response to hypoxia could trigger cellular dysregulation and/or placental vascular malformation. Further improvement of cell and animal models as well as in vivo imaging technologies to enable real-time detection of tissue oxygenation would deepen our understanding of the mechanisms of early placentation, provide early diagnostic detection and therapeutic treatment for the broad spectrum of pregnancy complications, and also improve assisted reproductive biology.

## Abbreviations

AMPK	Adenosine 5′ monophosphate-activated protein kinase
ARNT	Aryl hydrocarbon receptor nuclear translocator
Bcl-1	Beclin-1
BM	Bone marrow
CAT	Catalase
cNK	Circulating NK
CO	Carbon monoxide
CTBs	Cytotrophoblasts
DC-SIGN	Dendritic cell-specific intercellular adhesion molecule-3-grabbing non-integrin
dMϕ	Decidual macrophages
EGFR	Epidermal growth factor receptor
Elf5	E74-like factor 5
EPAS	Endothelial PAS domain protein
ER	Endoplasmic reticulum
ESCs	Embryonic stem cells
EVTs	Extravillous trophoblasts
Fe^2+^	Ferrous iron
FGR	Fetal growth restriction
GPx	Glutathione peroxidase
HBCs	Hofbauer cells
HCG	Human chorionic gonadotropin
Het	Heterozygous
HIF	Hypoxia-inducible factor
HLA-G	Human leukocyte antigen-G
HO-1	Heme oxygenase-1
HPL	Human placental lactogen
HRES	Hypoxic response elements
HSCs	Hematopoietic stem cells
IVF	In vitro fertilization
KO	Knockout
M1	Classically activated macrophages
M2	Alternatively activated macrophages
MDSCs	Myeloid-derived suppressor cells
MHC	Major histocompatibility complex
miRNAs	MicroRNAs
mTOR	Mammalian target of rapamycin
mTORC1	mTOR complex 1
mTORC2	mTOR complex 2
O_2_	Oxygen
PaO_2_	Partial pressure of arterial O_2_ (in mm Hg)
Plet1	Placenta expressed transcript 1
RAPTOR	Rapamycin-associated TOR protein
RICTOR	Rapamycin-insensitive companion of mTOR
ROS	Reactive oxygen species
sFlt-1	Soluble fms-like tyrosine kinase-1
SOD	Superoxide dismutase
STBs	Syncytiotrophoblasts
sVEGR1	Soluble VEGR receptor-1
TAMs	Tumor-associated macrophages
TLR	Toll-like receptor
trNK	Tissue resident NK
uNK	Uterine natural killer
VEGR	Vascular endothelial growth factor
VHL	von Hippel–Lindau protein
WT	Wild-type
